# Improving Access to Online Health Information With Conversational Agents: A Randomized Controlled Experiment

**DOI:** 10.2196/jmir.5239

**Published:** 2016-01-04

**Authors:** Timothy W Bickmore, Dina Utami, Robin Matsuyama, Michael K Paasche-Orlow

**Affiliations:** ^1^ Northeastern University College of Computer and Information Science Boston, MA United States; ^2^ Virginia Commonwealth University Richmond, VA United States; ^3^ Boston Medical Center Boston, MA United States

**Keywords:** embodied conversational agent, search user interface, information retrieval user interface, Web search, health literacy, relational agent, computer literacy, search engine, Internet

## Abstract

**Background:**

Conventional Web-based search engines may be unusable by individuals with low health literacy for finding health-related information, thus precluding their use by this population.

**Objective:**

We describe a conversational search engine interface designed to allow individuals with low health and computer literacy identify and learn about clinical trials on the Internet.

**Methods:**

A randomized trial involving 89 participants compared the conversational search engine interface (n=43) to the existing conventional keyword- and facet-based search engine interface (n=46) for the National Cancer Institute Clinical Trials database. Each participant performed 2 tasks: finding a clinical trial for themselves and finding a trial that met prespecified criteria.

**Results:**

Results indicated that all participants were more satisfied with the conversational interface based on 7-point self-reported satisfaction ratings (task 1: mean 4.9, SD 1.8 vs mean 3.2, SD 1.8, *P*<.001; task 2: mean 4.8, SD 1.9 vs mean 3.2, SD 1.7, *P*<.001) compared to the conventional Web form-based interface. All participants also rated the trials they found as better meeting their search criteria, based on 7-point self-reported scales (task 1: mean 3.7, SD 1.6 vs mean 2.7, SD 1.8, *P*=.01; task 2: mean 4.8, SD 1.7 vs mean 3.4, SD 1.9, *P*<.01). Participants with low health literacy failed to find any trials that satisfied the prespecified criteria for task 2 using the conventional search engine interface, whereas 36% (5/14) were successful at this task using the conversational interface (*P*=.05).

**Conclusions:**

Conversational agents can be used to improve accessibility to Web-based searches in general and clinical trials in particular, and can help decrease recruitment bias against disadvantaged populations.

## Introduction

The majority of US adults look online for health information [1,2]. However, disparities in the use of the Internet for finding health information remain [3,4]. One specific cause of these disparities may be that keyword-based search engines such as Google—although the primary search portals for most users—may actually represent a significant barrier for many disadvantaged individuals. Prior research has demonstrated that people with low health literacy, the ability to acquire and act on information related to health care [5,6], have particular difficulty using keyword-based search interfaces. Agree et al [3] demonstrated that individuals with low health literacy had lower success rates when using these interfaces to search for general health information on the Web. Usability by people with low health literacy is important because this population comprises 36% of US adults [5].

In addition to general-purpose search engines, many search engines and interfaces have been developed for specific kinds of health care information. One example is the clinical trial search engine, which retrieves descriptions of clinical trials from a repository or database [7]. Several of these search engines are available on the Web, developed by both commercial firms and the US government (eg, the National Cancer Institute [8]). Individuals use these search engines to find trials for which they may be eligible and in which they may be interested in participating. Utami et al [9] found that individuals with low health literacy found fewer clinical trials and took longer to complete standardized search tasks using a Web-based clinical trial search engine compared to those with adequate health literacy. Usability of clinical trial search engines by people with low health literacy is especially important because there is a disproportionate representation of minorities in this group [10-12] leading to reduced access for disadvantaged populations to information about clinical trials. Although Web-based clinical trial search engines hold the promise of providing universal access to information, conventional search systems may further promote disparities in clinical trial recruitment by catering primarily to populations of well-educated individuals with high levels of health and computer literacy.

Conventional Web form-based search engine user interfaces (eg, Google) typically make exclusive use of user-supplied keywords, whereas others combine keyword input with multiple-choice options, referred to as “facet-based” search interfaces [13]. Several prior studies have investigated the use of these search interfaces for users with low domain knowledge [14], who speak a language that is different than that of the Web form [15], who are children [16], or older adults [17], all who share characteristics with our task and population. These studies have demonstrated that even the simplest keyword-based search interfaces are unusable for many users and that special design considerations—such as simplifying results [17] and providing language and interaction support [16]—are important for disadvantaged users. Users may also be influenced by contextual cues when evaluating results from search engines [18] and those with low health literacy may be particularly susceptible to these cues when evaluating search results, relying on such features as position in search results, quality of pictures, and celebrity endorsements [19].

In this paper, we describe the design of a Web-based clinical trial search engine that we designed to mitigate barriers associated with low health literacy. The search task is framed as a conversation with an animated character to make it as familiar and approachable as possible, and a number of additional features and simplifications were made to help users with low health literacy navigate the overall clinical trial search process. We conducted a randomized trial, comparing the conversational search engine to an existing conventional Web-based search engine.

### Design of the Conversational Search Engine

The overall task the conversational search engine supports is finding one or more cancer-related clinical trials for which the user is eligible, based on initial demographic criteria and in which the user is provisionally interested, using publically available information. The search engine indexes trials from the National Cancer Institute’s (NCI) database of more than 10,000 active trials (at the time this work was conducted) [8].

**Figure 1:**
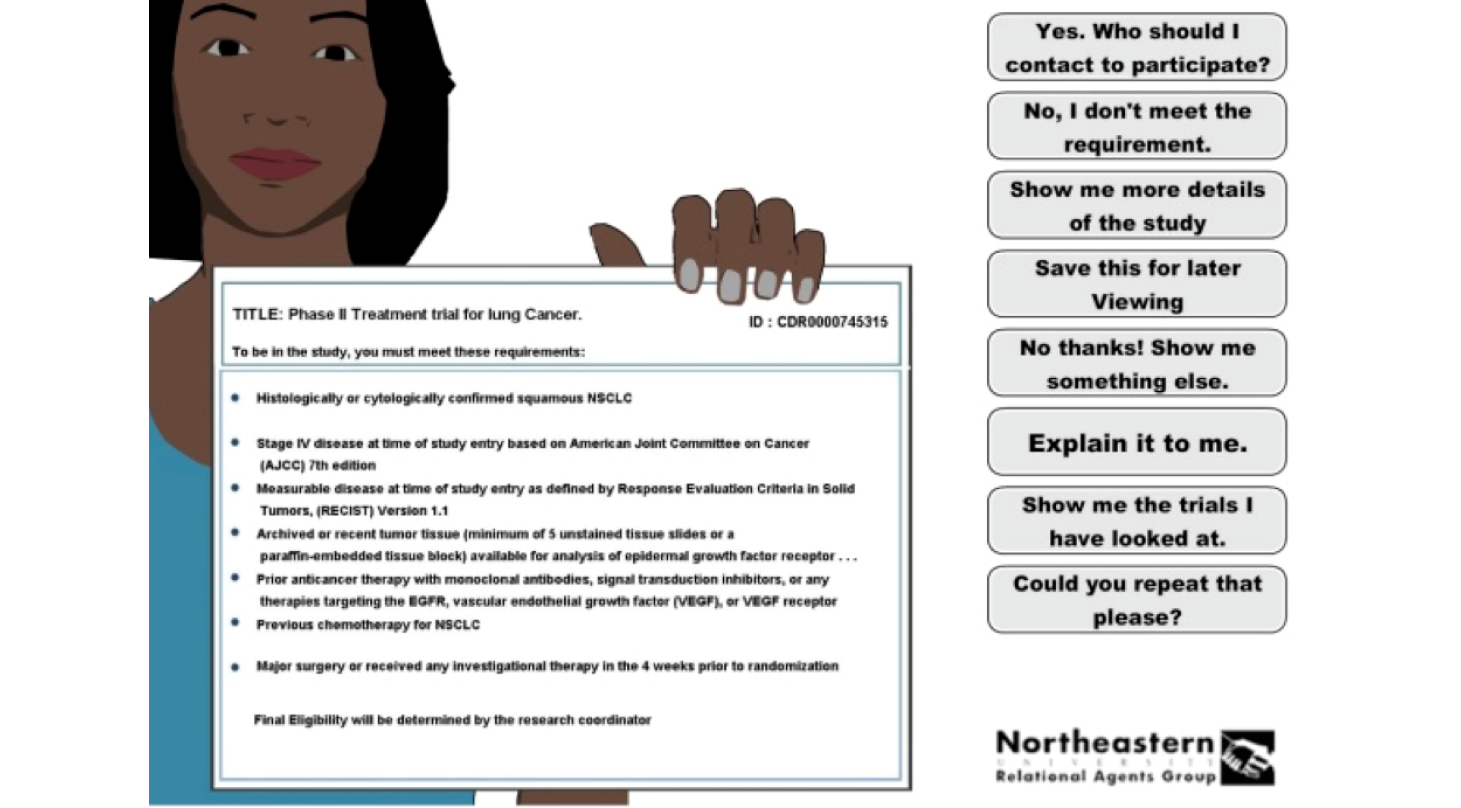
Conversational agent search interface.

Based on our experience in developing several health counseling dialog systems for patients with low health literacy [20], previous studies demonstrating greater user recall with audiovisual information combined with conversational style [21], and with animation combined with speech compared to text [22], we designed the overall interaction as a dialog with an embodied conversational agent [23]. The agent speaks using synthetic speech, generated from an augmented transition network-based dialog engine [24], template-based text generation [25], and a dynamically updated user model accompanied by conversational nonverbal behavior (eg, hand gestures, facial displays, gaze) animated in synchrony with the speech [26] (Figure 1). The agent also manipulates artifacts it is discussing with the user; in this case, documents that represent aspects of the clinical trials being discussed. User inputs to the conversation are restricted to multiple-choice selection of utterances from a list that is dynamically updated during each turn of the conversation. Thus, the interaction is system-initiated at the dialog adjacency-pair level (eg, agent question / user response), but user initiative is provided by allowing the user to select topics of conversation and ask questions at predefined points in the dialog by selecting from predefined lists. We have successfully used this interface modality with more than a thousand patients in clinical trials, including hundreds who have low health literacy and many who have never touched a computer before [20,27]. The resulting system could be characterized as a “fully faceted” search interface in which users are never asked to recall and type text, but are always scaffolded with the range of possible inputs they can make [13].

**Figure 2:**
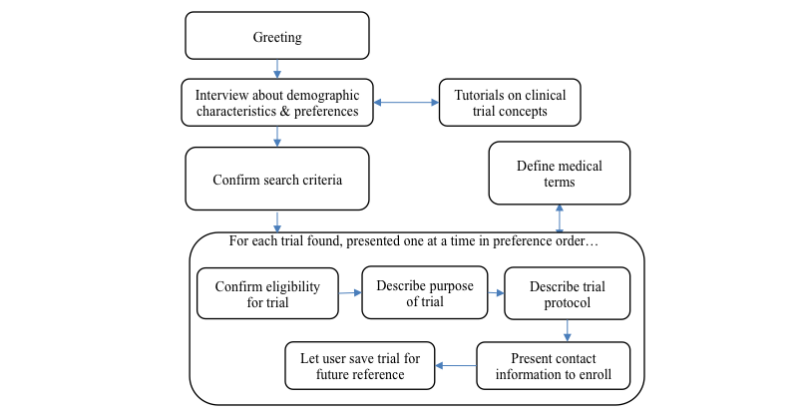
Typical dialog flow in a search session.

The overall search experience is framed as an extended conversation, in which the user is first interviewed about their requirements and preferences and then shown candidate trials with the agent providing as much scaffolding—through tutorials, explanations, and suggestions—along the way as possible. Given that clinical trial descriptions can be very complex and tedious for users to read, we err on the side of eliciting as much information as possible from users before the search in an attempt to identify trials that are most fitting. In addition, we designed the system to display information about a trial in stages, revealing only the details a user needs at each point of their evaluation. The overall flow of a typical conversation is shown in Figure 2.

To define the search criteria the agent elicits from the user, we leveraged qualitative findings from our usability study [9]. Participants in this study were asked to choose between pairs of clinical trial descriptions and then asked to explain their rationale. Analysis of explanations using grounded theory [28] revealed information-seeking practices and deliberation themes. We cross-referenced the resulting list of search criteria preferences elicited from users in this study with the clinical trial schema in the NCI database. We found that some user criteria already existed as database indexes, including participant age, sex, cancer type, study geographic location, trial type and phase, and the use of an investigational drug. Additional user criteria did not exist as database indexes, but could be inferred through text classification of text fields in the database, including subjective assessment of the likelihood that study participation would involve painful procedures, subjective assessment of protocol invasiveness (eg, survey vs diagnostic vs treatment), and overall time commitment. These inferred criteria (pain, invasiveness, and time burden) were computed for each trial based on a decision tree algorithm (ID3 [29]) that used word occurrence features in the trial description text trained on hand-rated examples. In our runtime clinical trial search algorithm, the search criteria that could be mapped to existing database indexes are used to search the database, whereas the criteria inferred via text classification are used to sort results.

In addition to the overall structure of the interaction shown in Figure 2, we included several features in the search user interface to assist users with low literacy in their search:

1. Dictionary: the NCI website provides users with a dictionary of medical terms; however, this dictionary is available on the NCI site as a separate module from the search engine. In our user studies of this website, we observed that finding definitions often distracted users from their main search task. We integrated a dictionary with our search functionality; although the agent explains search results to users, the character automatically extracts difficult terms from the text and offers to explain them.
2. Read aloud: users are able to ask the agent to read aloud, and repeat if necessary, any clinical trial text or definition. While speaking aloud, the agent holds up a visualization of the text, enabling users to read along.
3. Simplified title: clinical trial titles can be very long, complex, and hard to remember. We simplified display titles using the phase and type of the trial, and cancer type the trial is for (eg, replacing “A Study of BFTZ2252 Given With Patanobib or Dopepaxel in Subjects With Non-Small Cell Lung Cancer” with “Phase II Treatment Trial for Lung Cancer”).
4. Education modules: the agent offers to explain several concepts underlying clinical trials, such as voluntariness and risk, at appropriate points in the search.
5. User search criteria confirmation: the agent periodically displays the user’s search criteria and allows them to revise their choices. This is done after the initial interview, after each matching clinical trial is discussed, and whenever the agent runs out of trials to show the user.
6. Query refinement: if a search returns no results or the user exhausts the list of indexed trials, the agent suggests ways to modify the search criteria.
7. Bookmarking: users can save references to clinical trials for review in subsequent search sessions.
8. Summary of views: the agent periodically displays a list of trials viewed during the search session with bookmarked trials highlighted.
9. Levels of detail: each clinical trial description is presented in 3 levels of detail with associated displays. Users are initially only shown the title and eligibility criteria for a trial (see Figure 1), but are then given the option to view the trial research purpose, after which they are given the option of viewing details of the trial procedures.

### Study Aims

The overall aim of this work is to develop a Web-based search interface that is more usable by individuals with low health and computer literacy. We hypothesize that the conversational search interface will lead to greater search successes and higher levels of satisfaction compared to conventional keyword- and facet-based search engines for all users, but that the differences will be especially pronounced for individuals with low health literacy.

## Methods

To evaluate our system, we conducted a between-subjects randomized trial comparing our conversational agent search engine (“agent”) to the conventional facet- and keyword-based search engine (“control”) developed by the NCI ([7]) with both search engine interfaces indexing the same set of clinical trials. Participants were recruited from a pool of adult English-speaking cancer patients from across the literacy spectrum. Participants already had sociodemographic measures recorded and health literacy assessments completed for a prior study at Virginia Commonwealth University. The study protocol was approved by the Boston Medical Center and Virginia Commonwealth University IRBs and informed consent was obtained from all study participants. Health literacy was assessed using the Rapid Estimate of Adult Literacy in Medicine (REALM) [30]. Participants were split into adequate and inadequate health literacy groups using a REALM score of 9th grade as a cut-off as other authors have done [31-34].

We asked participants to perform 2 search tasks. In task 1, participants were asked to search for a clinical trial for which they would be provisionally eligible and in which they would be interested. In task 2, they were asked to search for a trial for someone else with specified eligibility criteria (ie, age, cancer type, trial type, geographic location) as a standardized test so that we could determine whether any trials they found actually matched the specified criteria (Textbox 1).

**Figure 3:**
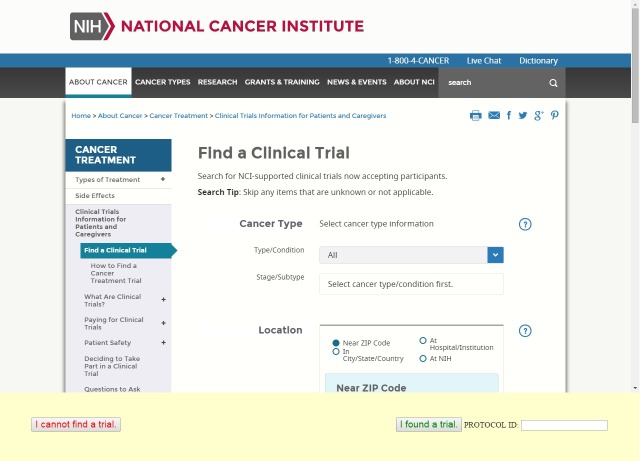
Experimental setup for control condition.

To ensure accessibility for participants with low computer and health literacy, we designed the experiment so that they were able to do the study either in the laboratory (for those without access to computers) or at home. The experiment software first gave participants a short tutorial on using the system, including a practice task. The first search task was then displayed. To complete each task, participants were redirected onto another Web page that had the agent or the NCI search engine (Figure 3). If the participant found a trial, they entered the clinical trial ID number into a text field and clicked an “I found a trial” button. If they could not find a trial, they clicked on an “I cannot find a trial” button. As soon as users completed each task, they were prompted to fill in a Web form questionnaire that captured the study measures. At the completion of each task, the first 7 questions in Table 1 were automatically administered via Web forms. Participants were also asked to recall the number of trials they examined and the number of these that met their criteria after each task. The Web server also captured the clinical trial ID that the participants found (if any) and the time needed to complete each task. At the completion of both tasks, the remaining 5 questions (questions 8-12 in Table 1) were administered verbally by a research assistant (for those conducting the study in person) or over the phone (for those conducting the study remotely). Participants completing the study at home did so on their own, without online assistance, and were called within 36 hours of completing the online tasks to obtain final outcome measures. Nonparametric statistics (chi-square tests for frequencies and Mann-Whitney *U* tests for all other measures) were used given the nature of the data and nonnormality of most distributions.

**Standardized search task (task 2).**

Now here is your second task. Please write it down.

This is Rosa. She is a cancer patient.

<IMAGE OF ROSA>

Here is some information about Rosa:

Age: 70 years old. 

Cancer type: Breast cancer.

Trial type she would like: Treatment trial.

Location of trial she would like: Can be anywhere.

We would like you to use the information above to find a clinical trial for Rosa.

Once you find a trial, please enter its ID number into the box on the bottom right of the screen where it says "TRIAL ID” and click the button that says "I found a trial.” Also, please write down the protocol ID number on a piece of paper.

If you have spent some time looking but do not think you can find a trial, then click on the button that says "I cannot find a trial” at the bottom left corner of the screen.

If you are ready to begin, click the "I am ready" button.

**Table 1:** Self-report measures used in the study.

Scale self-report item	Anchor 1	Anchor 7
1. To what degree did you know what you wanted in a trial? (task 1)	Not at all	Exactly
2. To what degree did the trial match what you were looking for?	Not at all	Exactly
3. How likely are you to sign up for the trial that you found? (task 1)	Not likely	Very likely
4. How much time do you feel it took to use the system?	Too little	Too much
5. How satisfied were you with the clinical trial search system?	Not at all	Very
6. How frustrated do you feel right now?	Not at all	Very
7. How pleased do you feel right now?	Not at all	Very
8. How much pressure did you feel to volunteer for a trial?	No pressure	A lot of pressure
9. How much information do you feel was presented by the system?	Not enough	Too much
10. How likely would you be to use the system again, if you wanted to find another trial?	Not likely	Very likely
11. How likely would you be to recommend the system to someone else who was looking for a trial?	Not likely	Very likely
12. How much do you trust the information you received from the system?	Not at all	Very much

## Results

Participant sociodemographic information is shown in Table 2. A total of 89 individuals participated; mean age was 59.2 (SD 9.8) years, 46% (48/89) were female, and 27% (23/89) had low health literacy. A current cancer diagnosis was reported by 98% (87/89) of participants: 32% (28/89) with hematologic cancer, 14% (12/89) with breast cancer, 14% (12/89) with genitourinary cancer, 14% (12/89) with head and neck cancer, and 10% (9/89) with lung cancer. Most (70%, 62/89) reported regular computer use and regular use of Web-based search engines (52%, 46/89). Although only 21% (19/89) reported previous participation in a cancer-related clinical trial, 52% (46/89) expressed interest in participating in one. Approximately half of the participants (48%, 43/89) were randomized to the agent condition. Of the 89 participants, 53 (60%) conducted the study in the laboratory and 36 (40%) conducted the study over the Web at home. A few participants could not complete some of the tasks (17%, 14/89) due to technical or other problems. The primary study results are shown in Table 3.

### Task 1 Results for All Participants

In the initial task, participants were asked to find a clinical trial for themselves. Most participants started this task without a clear idea of what they were looking for and rated the degree they knew what they wanted in a trial a mean 2.8 (SD 1.9) on a scale of 1 to 7. Nevertheless, 45% (19/42) in the agent group and 31% (14/45) in the control group successfully found a trial; there was no significant difference between groups (χ^2^_1_=1.8, *P*=.52). The degree to which participants felt these trials matched what they were looking for was significantly greater in the agent condition compared to the control condition (mean 3.7, SD 1.6 vs mean 2.7, SD 1.8, *U*=465, *P*=.01). Participants were significantly more satisfied with the agent compared to the conventional interface (rating mean 4.9, SD 1.8 vs mean 3.2, SD 1.8, *U*=363, *P*<.001) and felt significantly less frustrated (rating mean 2.1, SD 1.7 vs mean 3.7, SD 2.2, *U*=405, *P*<.001) and more pleased (rating mean 5.1, SD 2.1 vs mean 3.4, SD 1.9, *U*=380, *P*<.001) with the agent after completing the task compared to those in the control condition.

There were no significant differences between results for those who completed the study in person versus at home.

### Task 2 Results for All Participants

In the second task, participants were asked to find a clinical trial that satisfied a prespecified set of criteria as a standardized task. Although 48% (20/42) in the agent group and 40% (18/45) in the control group claimed to find trials that met the criteria (χ^2^_1_=0.5, *P*=.52), only 43% (18/42) and 31% (14/45), respectively, actually found a correct trial (χ^2^_1_=1.3, *P*=.28). However, participants in the agent group felt that the trials they found matched the criteria to a greater degree compared to those in the control group (mean 4.8, SD 1.7, vs 3.4, SD 1.9, *U*=381, *P*<.001). As with task 1, participants in the agent group were significantly more satisfied (rating mean 4.8, SD 1.9 vs mean 3.2, SD 1.7, *U*=336, *P*<.001) and pleased (rating mean 4.6, SD 1.8 vs mean 3.1, SD 1.7, *U*=358, *P*<.001), and significantly less frustrated (rating mean 2.6, SD 1.9 vs mean 3.8, SD 2.2, *U*=429, *P*=.01) after completing their task compared to those in the control group.

As with task 1, searching with the agent tended to take longer compared to the conventional interface (mean 8.2, SD 5.3 minutes vs mean 6.4, SD 4.3 minutes), but this did not meet statistical significance (*U*=507, *P*=.06). However, participants felt the agent took significantly less time compared to the conventional interface (mean 4.2, SD 1.1 vs 5.1, SD 1.7, *U*=466, *P*=.03).

**Table 2:** Participant sociodemographics.

Variable		All N=89	Agent n=43	Control n=46	*P*
Sex (female), n (%)		48 (54)	18 (42)	30 (65)	.03
Age (years), mean (SD)		59.2 (9.8)	58.6	59.7	.59
**Race, n (%)**					.45
	Black	41 (46)	19 (44)	22 (48)	
	White	48 (54)	24 (56)	24 (52)	
	Other	0 (0)	0 (0)	0 (0)	
Hispanic or Latino, n (%)		0 (0)	0 (0)	0 (0)	N/A
**Education, n (%)**					.38
	<High school	15 (17)	9 (21)	6 (13)	
	High school	10 (11)	6 (14)	4 (9)	
	>High school	62 (71)	27 (63)	35 (78)	
Married, n (%)		40 (45)	16 (32)	24 (52)	.20
**Health literacy (REALM Score)**					
	Mean (SD)	57 (15)	56 (16)	59 (15)	.35
	Adequate (≥60), n (%)	65 (73)	27 (64)	38 (83)	.05
Study location (in person), n (%)		53 (60)	27 (63)	26 (57)	.67
**Computer experience, n (%)**					.64
	Never used one	7 (8)	5 (12)	2 (4)	
	Tried one	21 (24)	10 (24)	11 (24)	
	Use regularly	53 (61)	24 (57)	29 (64)	
	Expert	6 (7)	3 (7)	3 (7)	
**Search engine experience, n (%)**					.99
	Never used one	15 (17)	7 (17)	8 (18)	
	Tried one	16 (18)	8 (19)	8 (18)	
	Use regularly	45 (52)	22 (52)	23 (51)	
	Expert	11 (13)	5 (12)	6 (13)	
**Clinical trials knowledge, n (%)**					.12
	None	16 (18)	12 (29)	4 (9)	
	A little	40 (46)	16 (38)	24 (53)	
	Fair amount	29 (33)	13 (31)	16 (36)	
	Expert	2 (2)	1 (2)	1 (2)	
Participated in cancer clinical trial before (yes), n (%)		18 (21)	7 (17)	11 (24)	.43
Actually interested in participating in a trial now? (yes), n (%)		44 (52)	23 (56)	21 (48)	.52
To what degree do you know what you want in a trial?^a^ mean (SD)		2.8 (1.9)	2.7 (1.7)	3.0 (2.1)	.60

^a^ Anchor 1=I didn’t know at all; anchor 7=I knew exactly.

### Results for Low Health Literacy Participants

Table 4 shows the results by study condition for the 24 participants with low health literacy. The results are very similar to those for all study participants (Table 3), with one notable exception: in the standardized task (task 2), none of the low literacy participants were able to find a clinical trial that met the given criteria using the conventional interface. However, 36% (5/14) of low literacy participants were able to find a correct clinical trial using the agent. This difference was near significant (χ^2^_1_=3.7, *P*=.05).

**Table 3:** Primary study results.

Measure	Task 1			Task 2		
	Agent (n=43)	Control (n=46)	*P*	Agent (n=43)	Control (n=46)	*P*
Completed task, n (%)^a^	37 (86)	37 (80)	.58	36 (84)	37 (80)	.79
Declared found a trial, n (%)^a^	19/42 (45)	14/45 (31)	.19	20/42 (48)	14/45 (40)	.52
Found a correct trial, n (%)^a^	—^b^	—^b^	—^b^	18/42 (43)	14/45 (31)	.28
Elapsed time (minutes), mean (SD)^c^	12.6 (9.2)	9.0 (8.4)	.06	8.15 (5.3)	6.4 (4.9)	.06
Number of trials examined (self-report), mean (SD)^c^	2.8 (3.0)	3.8 (6.7)	.56	3.0 (3.1)	4.9 (9.4)	.54
Trials examined that meet criteria (self-report; % of trials examined), mean (SD)^c,d^	56 (39)	34 (35)	.06	64 (37)	44 (41)	.09
To what degree did the trial match what you were looking for? (range 1-7),^c^ mean (SD)	3.7 (1.8)	2.7 (1.6)	.01	4.8 (1.7)	3.4 (1.9)	.00
How likely are you to sign up for the trial that you found? (range 1-7), mean (SD)^c^	3.3 (1.7)	2.9 (1.7)	.21	—^e^	—^e^	—^e^
How much time do you feel it took to use the system? (range 1-7), mean (SD)^c^	4.3 (1.3)	4.6 (1.8)	.61	4.2 (1.1)	5.1 (1.7)	.03
How satisfied were you with the clinical trial search system? (range 1-7), mean (SD)^c^	4.9 (1.8)	3.2 (1.8)	<.001	4.8 (1.9)	3.2 (1.7)	<.001
How frustrated do you feel right now? (range 1-7), mean (SD)^c^	2.1 (1.7)	3.7 (2.2)	.001	2.6 (1.9)	3.8 (2.2)	.01
How pleased do you feel right now? (range 1-7), mean (SD)^c^	5.1 (2.1)	3.4 (1.9)	.001	4.6 (1.8)	3.1 (1.7)	.001
How much pressure did you feel to volunteer for a trial? (range 1-7), mean (SD)^c^	1.2 (0.6)	1.4 (1.1)	.99	—^e^	—^e^	—^e^
How much information do you feel was presented by the system? (range 1-7), mean (SD)^c^	4.2 (1.7)	4.3 (1.8)	.98	—^e^	—^e^	—^e^
How likely would you be to use the system again, if you wanted to find another trial? (range 1-7), mean (SD)^c^	5.0 (2.1)	4.1 (2.4)	.07	—^e^	—^e^	—^e^
How likely would you be to recommend the system to someone else who was looking for a trial? (range 1-7), mean (SD)^c^	5.1 (2.2)	4.5 (2.5)	.25	—^e^	—^e^	—^e^
How much do you trust the information you received from the system? (range 1-7), mean (SD)^c^	5.7 (1.6)	5.1 (1.9)	.13	—^e^	—^e^	—^e^

^a^ Chi-square test.

^b^ Task 1 involved participants finding trials they were interested in, so there was no way to objectively assess whether the trials they found were “correct”.

^c^ Mann-Whitney *U* test.

^d^ Trials examined that meet criteria was a subjective self-report measure.

^e^ Task 2 involved participants finding trials to satisfy criteria for a hypothetical patient, so it did not make sense to ask questions related to their own participation.

**Table 4:** Study results for low health literacy participants.

Measure	Task 1			Task 2		
	Agent (n=15)	Control (n=8)	*P*	Agent (n=15)	Control (n=8)	*P*
Completed task, n (%)^a^	12 (80)	8 (100)	.18	12 (80)	8 (100)	.18
Declared found a trial, n (%)^a^	6/14 (43)	1/8 (13)	.14	5/14 (36)	1/8 (13)	.24
Found a correct trial, n (%)^a^	—^b^	—^b^	—^b^	5/14 (36)	0/8 (0)	.05
Elapsed time (minutes), mean (SD)^c^	13.3 (11.4)	8.2 (6.3)	.47	6.8 (4.3)	4.6 (4.3)	.25
Number of trials examined (self-report), mean (SD)^c^	3.0 (3.4)	0.9 (1.1)	.16	2.8 (3.2)	1.5 (2.1)	.36
Trials examined that meet criteria (self-report; % of trials examined), mean (SD)^c,d^	74 (30)	75 (35)	.89	80 (31)	67 (58)	.92
To what degree did the trial match what you were looking for? (range 1-7), mean (SD)^c^	4.1 (1.9)	2.4 (1.4)	.06	5.3 (2.1)	3.3 (2.0)	.04
How likely are you to sign up for the trial that you found? (range 1-7), mean (SD)^c^	3.8 (1.1)	3.6 (1.9)	.81	—^e^	—^e^	—^e^
How much time do you feel it took to use the system? (range 1-7), mean (SD)^c^	3.8 (0.9)	4.1 (2.5)	.83	3.9 (0.3)	4.5 (2.3)	.86
How satisfied were you with the clinical trial search system? (range 1-7), mean (SD)^c^	5.3 (1.6)	2.9 (1.7)	.01	5.7 (1.6)	2.9 (1.4)	.002
How frustrated do you feel right now? (range 1-7), mean (SD)^c^	2.9 (2.0)	4.8 (2.1)	.01	2.3 (2.1)	3.1 (1.9)	.15
How pleased do you feel right now? (range 1-7), mean (SD)^c^	5.5 (1.6)	3.3 (2.7)	.04	5.7 (1.7)	2.6 (1.1)	.001
How much pressure did you feel to volunteer for a trial? (range 1-7), mean (SD)^c^	1.1 (0.3)	2.3 (2.2)	.10	—^e^	—^e^	—^e^
How much information do you feel was presented by the system? (range1-7), mean (SD)^c^	4.8 (1.5)	4.3 (1.5)	.21	—^e^	—^e^	—^e^
How likely would you be to use the system again, if you wanted to find another trial? (range 1-7), mean (SD)^c^	5.5 (1.8)	5.0 (2.4)	.74	—^e^	—^e^	—^e^
How likely would you be to recommend the system to someone else who was looking for a trial? (range 1-7), mean (SD)^c^	6.1 (1.9)	5.1 (2.7)	.45	—^e^	—^e^	—^e^
How much do you trust the information you received from the system? (range 1-7), mean (SD)^c^	6.3 (1.0)	5.1 (2.1)	.71	—^e^	—^e^	—^e^

^a^ Chi-square test.

^b^ Task 1 involved participants finding trials they were interested in, so there was no way to objectively assess whether the trials they found were “correct”.

^c^ Mann-Whitney *U* test.

^d^ Trials examined that meet criteria was a subjective self-report measure.

^e^ Task 2 involved participants finding trials to satisfy criteria for a hypothetical patient, so it did not make sense to ask questions related to their own participation.

Analyzing differences between low and high health literacy participants across all study conditions indicated a few significant differences. Participants with low health literacy were more likely overall to state that the trials they read satisfied their criteria compared to those with high health literacy (task 1: 74% vs 37%, *U*=82, *P*<.001; task 2: 77% vs 49%, *U*=148, *P*=.02). Participants with low health literacy were more likely to say they would sign up for the trial they found in task 1 (rating mean 3.8, SD 1.4 vs mean 2.8, SD 1.8, *U*=380, *P*=.048) and that they would recommend the system to a friend (rating mean 5.7, SD 2.3 vs mean 4.5, SD 2.3, *U*=379, *P*=.01) compared to those with adequate health literacy.

## Discussion

### Principal Results

In our comparison of a conversational agent-based search user interface to a conventional keyword- and facet-based search engine interface, participants were more satisfied with the agent and felt the agent was better at finding trials that matched their criteria compared to the conventional interface. Participants also felt more pleased and less frustrated after interacting with the agent compared to the conventional interface.

In our standardized task (task 2), it is notable that none of the low health literacy participants were able to find a correct clinical trial using the conventional search engine interface, whereas 36% (5/14) were able to do so with the conversational agent. These results reinforce our earlier findings that conventional search interfaces are unusable by individuals with low health or computer literacy [9]. It is encouraging that the conversational interface was able to provide accessibility to at least a third of these users, while being rated more highly on satisfaction by all users, including those with high health literacy. Nonetheless, it appears that research on additional adaptations is warranted in order to succeed with an even broader portion of the population.

The conversational interface does take more time to use compared to the conventional interface: 40% longer in task 1 and 27% longer in task 2 (although these differences were not statistically significant). There are several reasons for this: the time required to hear spoken prompts rather than reading them, the interview by the agent to obtain search criteria, and social dialog, tutorials, and other “off-task talk” used by the agent to improve approachability, engagement, and comprehension. However, users in our target demographic are clearly happy to spend the extra time with the conversational user interface to obtain better results; in the browsing task, they chose to spend almost twice as long finding a trial compared to those using the conventional interface. In addition, their subjective impression of time taken in task 2 indicates that users felt the conversational agent interface actually took significantly less time to use compared to the conventional interface.

### Limitations

Our study had several limitations beyond the small number of participants involved. Some (21%) of the users in our study had previously been involved in clinical trials and thus are not representative of the general population of people with cancer because they may have had higher than average background knowledge about clinical trials. However, people with prior trial experience were randomly assigned to the 2 trial arms, so both groups should be equivalent in this regard. Another limitation relates to the use of the REALM as a measure of health literacy. Although this tool successfully differentiated among participants regarding the impact of our intervention, other measures could potentially have provided a more refined capacity to delve deeper within specific dimensions of electronic and computer literacy. Although the eHealth Literacy Scale (eHEALS) was designed for such a purpose, it is a subjective self-report measure [35]. We opted for an objectively scored measure in the current study; future research is warranted to further differentiate how a conversational search interface may ameliorate various dimensions of low health and computer literacy.

### Comparison With Prior Work

Several studies have investigated the use of standard keyword-based search interfaces for users with low domain knowledge [14], who speak a foreign language [15], who are children [16] or older adults [17], which all share characteristics with our task and population. These studies have demonstrated that even the simplest keyword-based search interfaces are unusable for many users and that special design considerations—such as simplifying results [17] and providing language and interaction support [16]—are important for users, especially those with low health or computer literacy

Other studies have investigated the use of conversational agents to communicate health information to individuals with low health literacy. Bickmore et al [20,36] and Wang et al [37] have developed conversational agents for physical activity promotion, hospital discharge instruction, explanation of medical documents, and family health history-taking to individuals with low health literacy. Most of these studies have demonstrated that participants with low health literacy have significantly higher levels of satisfaction with conversational interfaces compared to participants with adequate health literacy.

### Conclusions

Our findings suggest that conversational agent-based search engine interfaces could be a good alternative to conventional Web form-based interfaces for many kinds of applications, but especially for those intended for low health literacy users or those with limited computer experience or skills.

## Abbreviations

NCI: National Cancer Institute
REALM: Rapid Estimation of Adult Literacy in Medicine
